# How health warning labels on wine and vodka bottles influence perceived risk, rejection, and acceptance

**DOI:** 10.1186/s12889-022-12564-8

**Published:** 2022-01-24

**Authors:** Cornelia Staub, Michael Siegrist

**Affiliations:** ETH Zürich, Institute for Environmental Decisions, Consumer Behavior, Zürich, Switzerland

**Keywords:** Wine, Warning labels, Risk perception, Alcohol, Cancer

## Abstract

**Background:**

Wine consumption has a particular place in the culture of many European countries, and beliefs that wine offers health benefits are widespread. High consumption of wine and other alcoholic beverages among many Europeans correlates with alcohol-related accidents and disease burdens. Health warning labels (HWLs) on alcohol containers have been increasingly recommended to deter consumers from drinking. However, findings on the impact of HWLs on consumers’ behavior have been mixed. Moreover, many European consumers have been found to reject the use of warning labels as a policy intervention, especially for wine, perhaps due to its cultural and economic importance.

**Methods:**

An online study with a between-subjects design was conducted in Switzerland (*N* = 506) to assess whether HWLs can influence the perceived risk associated with drinking wine and vodka, a beverage insignificant to Swiss culture. Participants were presented an image of either a wine or vodka bottle with or without an HWL presenting a liver cancer warning statement. They were then asked to indicate their perceived risk of regularly consuming the depicted beverage. Acceptance and rejection of HWLs were also assessed.

**Results:**

The perceived risk of vodka consumption exceeded the corresponding risk for wine but was unaffected by an HWL. Perceived health benefits were the main, negative predictor of perceived consumption risk. Participants mainly rejected HWLs due to their perceived effectiveness, perceived positive health effects, social norms, and individualistic values.

**Conclusions:**

Perceived risk is an important determinant of drinking behavior, and our results suggest that HWLs may be unable to alter risk perceptions. Furthermore, a strong belief in the health benefits of alcohol consumption, particularly wine consumption, reduce risk perceptions and may be unaffected by HWLs.

## Introduction

The production of alcoholic beverages is an important sector in many European economies, and wine, beer, and spirits are central to many European cultures. In Switzerland, wine and beer are the most popular alcoholic beverages, whereas spirits such as vodka account for only a minor share of consumption [1]. Per capita, European citizens—including Swiss—consume twice as much alcohol as the world average [2]. It is thus unsurprising that Europeans’ norms of drinking alcohol correlate with alcohol-related diseases, accidents, and dependency [3].

Extensive literature has demonstrated alcohol’s harmfulness in terms of health risks (for a summary, see, e.g., [4]). However, several studies have reported that only a minority of consumers are aware of the threat that regular or excessive alcohol consumption poses to their health, such as cancer risk, and they are unfamiliar with recommended drinking guidelines to reduce such risks [5–7]. Risk perception is an important factor in determining the degree to which someone engages in a risky behavior, such as alcohol consumption [8, 9]. Rehm, Lachenmeier [10] suggested that the lack of knowledge about alcohol’s adverse effects has led to a high level of risk acceptance. Moreover, they suggested that educating people about the risks of alcohol consumption can change drinking behavior.

Interventions to increase consumers’ knowledge about alcohol have faced several challenges, however. First, previous research has found that consumers believe themselves to be well informed about alcohol’s health risks [11]. Therefore, they may not feel that they need more information about these risks. Second, young consumers especially often feel that they are not susceptible to long-term risks such as cancer, which may reduce the effectiveness of alcohol-risk information campaigns that communicate cancer risk [12]. Third, many consumers have been found to believe that wine offers beneficial health effects if consumed in moderation [7] and that other factors—such as genetic predisposition—may more decisively lead to cancer than lifestyle choices [13]. Such pre-existing beliefs are particularly widespread among people who consume large amounts of alcohol [14], which presents a challenge for the effectiveness of consumer-information interventions. In countries such as Italy or France, where drinking wine is part of social norms [15], it has been found that consumers associate adverse outcomes with other alcoholic beverages but not with wine [16].

The dilemma of alcohol consumption is often compared to the problems of smoking. Many researchers have suggested that instead of informing people about the risks of alcohol with information campaigns, consumption-deterring warning labels—such as the labels that have proven effective on tobacco products—may be a more promising, low-cost approach to decrease alcohol consumption [5, 17, 18]. Several countries have already introduced some sorts of health warning labels (HWLs) on alcohol containers, such as the United States, Australia, and France. These labels have repeatedly proven ineffective since consumers do not notice them due to their size and position or have remained unchanged after their implementation [19, 20]. Consequently, several studies have tested whether more salient, tobacco-like HWLs effectively deter consumers and affect drinking behavior. Front labels including warning text or even frightening images can increase consumers’ fear or negative emotions [13, 17]. Clarke, Pechey [21] reported that cancer warning statements increase consumers’ cancer risk perception but also increase their reactance to or rejection of such labels. Staub, Fuchs [22] found that HWLs increase wine-consumption risk perception whether they are only textual or use both text and images—but only if these HWLs do not specify consumption quantities. Other studies have found that such labels do not change alcohol-related outcome expectancies [23] and are unlikely to change behavior since these labels’ effects depend on whether consumers feel susceptible to the presented risks in the first place [24].

Moreover, the HWLs that most affected fear or consumption intentions also provoked the strongest reactance [21, 25]. Furthermore, few consumers perceived HWLs as acceptable, especially for wine [26, 27]. Rehm, Lachenmeier [10] argued that this lack of acceptance of and rejection of HWL is likely due to a lack of knowledge about alcohol’s risks. Furthermore, they suggested that other factors influence reactions to warning labels. One such factor may be attitudes toward alcohol and alcohol-related behaviors, which differ between cultures [12].

Many studies have used different alcoholic beverages to assess HWLs’ effect but have not reported on any differences in outcome variables between beverage types [21, 23, 27, 28]. Annunziata, et al. [29] suggested that consumers’ receptiveness of warning labels depends on the type of beverage. For example, warnings on beer were accepted more than warnings on wine. Moreover, Annunziata, Agnoli [16] stressed that future research should investigate beverage types’ role on reactions to HWLs and whether wine’s cultural role in many European countries causes consumers’ lack of HWL acceptance. Thus, HWLs on wine may be less accepted than HWLs on other alcoholic beverages, such as vodka, which is not as deeply rooted in western European cultures and economies as wine.

The effectiveness of interventions such as HWLs in changing drinking behavior varies between individuals. For example, consumers who consume large amounts of alcohol were found to have lower risk perceptions for alcohol consumption [14], so HWLs may be less effective for these consumers. Furthermore, the reaction to and acceptance of government interventions are also affected by personal opinions of governments’ roles in restricting individual rights to protect citizens, which are called *individualistic values* [30]. Staub, Fuchs [22] found that individualistic values are a major determinant of consumers’ acceptance of HWLs on wine bottles.

The present paper aims to address the following gaps in the literature. First, this study assesses HWLs’ potential to alter the perceived risk of alcohol consumption among a sample of Swiss consumers. Second, the study aims to investigate whether the HWLs’ effects on perceived risk and the acceptance of HWLs differ for alcoholic beverages with varying cultural significance to Swiss consumers, particularly wine and vodka.

We hypothesized that the perceived risk of regular alcohol consumption is higher among people exposed to HWLs on alcohol containers than people who are not exposed to HWLs. Based on widespread health beliefs about wine, we also assumed that the perceived risk of wine consumption is lower than the perceived risk of vodka consumption. Due to wine’s cultural significance in Switzerland, we hypothesized that HWLs on wine are perceived as less acceptable than HWLs on vodka. Furthermore, we sought to estimate how such factors as drinking norms, individualistic values, and alcohol consumption influence the rejection of HWLs on wine and vodka. Our results provide insights into the HWLs’ potential to increase consumers’ risk perceptions and, therefore, affect drinking behavior. Additionally, the present study adds to the knowledge of alcohol HWLs and how factors other than the labels themselves influence consumers’ HWL perceptions and reactions.

## Material and methods

To assess how HWLs on different beverages affect risk perceptions among alcohol consumers, we conducted an experiment. First, participants’ wine and spirits consumption frequencies and quantities were assessed. Then, participants were assigned to one of four experimental groups. Two groups were presented with an image of a wine bottle, while the other two groups were presented with an image of a vodka bottle. One wine bottle and one vodka bottle included an HWL depicting a statement about cancer risk, while the other bottles did not include HWLs. Participants were asked to state their perceived consumption risk. Next, all participants were presented with an image of a bottle of the beverage they had been presented in the previous step that included an HWL. We measured their acceptance, rejection, and perceived effectiveness of the depicted HWL. Further, we assessed participants’ perceived social norms of wine and vodka consumption, respectively, as well as their perceived positive health effects and perceived benefits of alcohol consumption generally. Finally, we used a scale by Kahan, Jenkins-Smith [30] to measure participants’ individualistic values, which may be relevant to HWL implementations on alcohol containers. The following subsections provide more detailed information about the various parts of this study.

### Data collection and sample characteristics

We conducted an online experiment with participants from the German-speaking part of Switzerland (*n* = 506). A market research company (Respondi AG) collected the study’s data in May 2021 until attaining the desired number of responses and meeting quotas for respondents’ age and gender. There were 50% men in the study sample, and the distribution of participants across the age groups was representative of the residential population in Switzerland with 37.4% in the group of 20 – 39 year-old age group, 46.4% in the group of 40 – 64 year-old age group, and 16.2% in the older than 65 year-old age group [31]. To be eligible to participate in the study, respondents had to be at least 18 years old and drink alcohol. We used the time taken to complete the questionnaire to exclude participants who seemed not to have taken time to carefully read and respond to our questions. Accordingly, participants whose completion times were below half of the median (*mdn* = 357 s) were excluded. In stating their gender, participants had the option to select *male*, *female*, or *other / not specified.* Only one respondent chose the latter option, which was an insufficient amount for statistical tests; therefore, this respondent was excluded. The study’s final sample comprised 251 male participants and 255 female participants. Their mean age was 47 years (*SD* = 16).

### Alcohol consumption

**Fig. 1:**
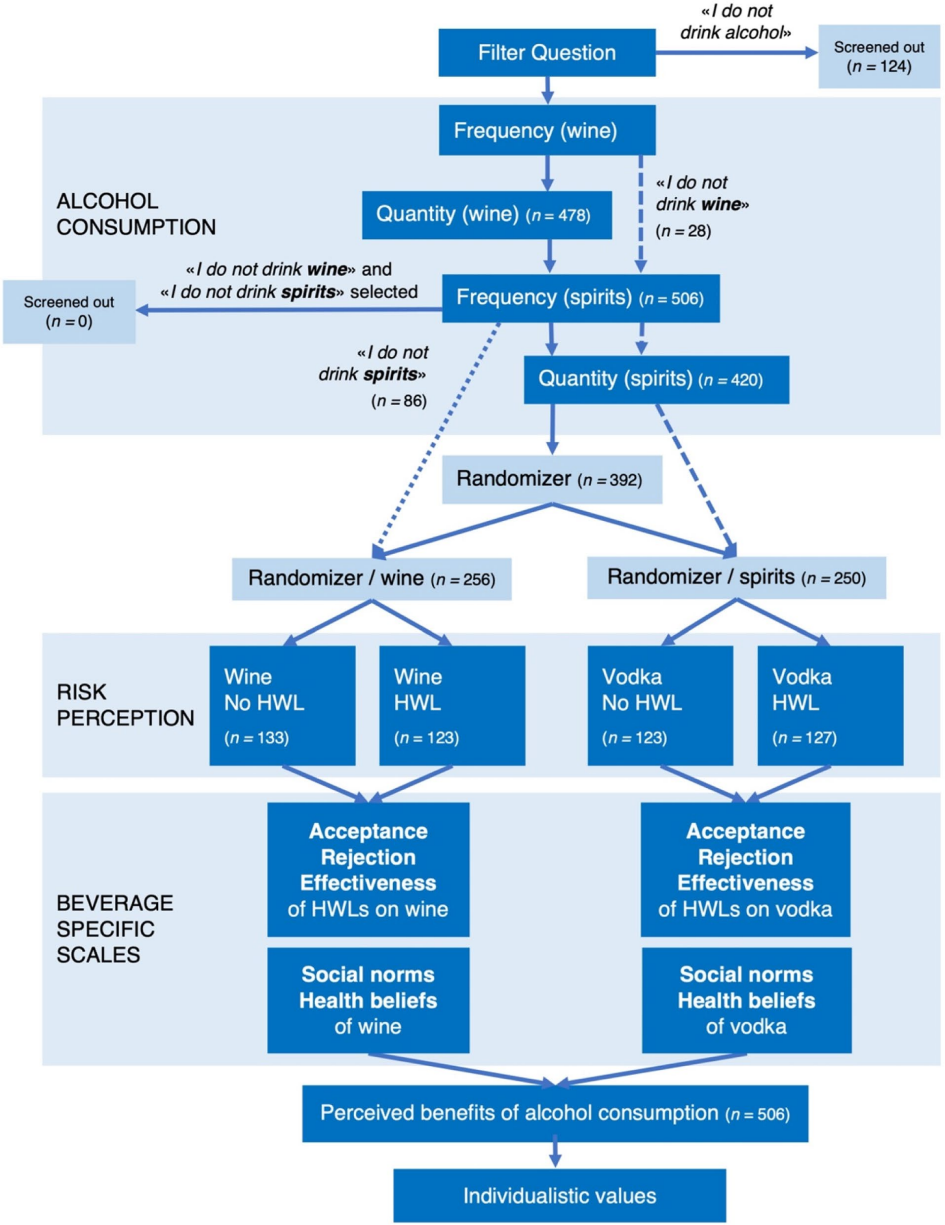
Procedure of questionnaire with group assignment according to alcohol consumption

Respondents were asked how often they consumed alcohol so that we could exclude respondents who did not drink alcohol. Then, respondents were asked how often they drank wine and how many 100 ml glasses they drank per occasion. In our analysis, we estimated numbers of standard glasses by multiplying consumption frequencies by consumption quantities. The answer options for consumption frequency were coded based on numbers of occasions per month: *I do not drink wine* (0), *less than once a month* (0.5), *about once a month* (1), *several times a month* (2), *about once a week* (4), *several times a week* (12), and *daily* (30). The answer options were recoded into specific numbers of glasses: *less than a glass* (0.5), *1–2 glasses* (1.5), *2–3 glasses* (2.5), *3–4 glasses* (3.5), *4–5 glasses* (4.5), *1 bottle* (7.5), or *more than 1 bottle* (8). Respondents’ total wine consumption, thus, represented an approximation of their number of standard units^1^ of wine consumed in one month. If someone stated not to drink wine, the person was directed to the questions about consumption frequency and quantity of spirits (see Fig. 1). The answer options for spirits consumption frequency were the same, and they were coded in the same manner. For spirits consumption quantities, participants were asked how many 40 ml glasses of spirits they normally drank per occasion. The answer options were the same as for wine, except that the highest possible quantities were *5–6 glasses* and *more than 6 glasses*, which were coded as 5.5 and 7 glasses, respectively. Again, a multiplication of respondents’ consumption frequency by quantity was used to estimate the number of standard units of spirits they had consumed per month. Participants who selected “I do not drink spirits” were not asked about their consumption quantity of spirits. If a participant answered that they drank neither wine nor spirits, they were excluded from the study since we sought to assess consumers of wine and spirits (Fig. 1).

### Experimental procedure

**Fig. 2:**
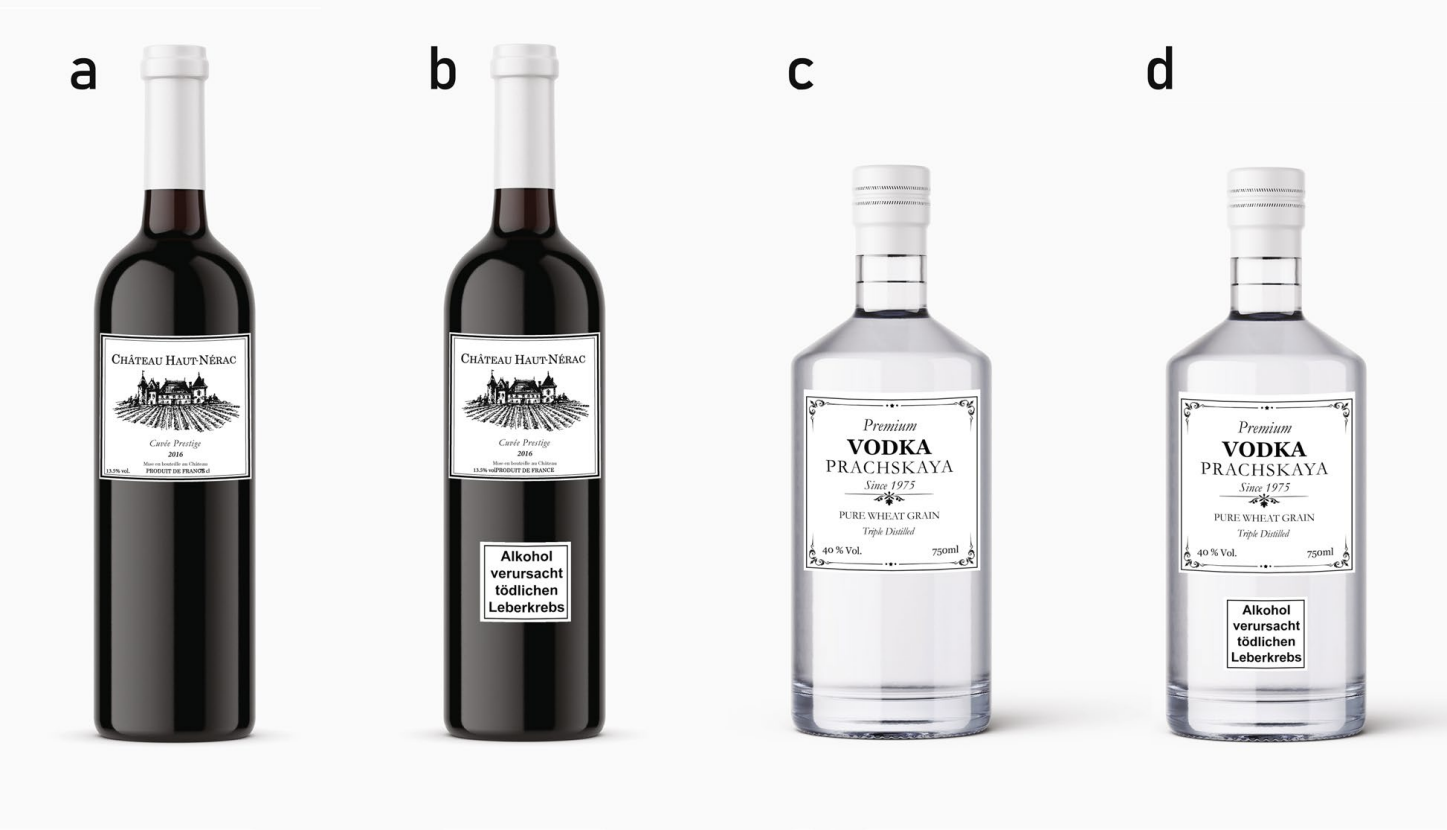
Alcohol bottle images presented to the four experimental groups

This study aimed to assess whether HWLs affect consumers’ alcohol risk perception and whether this effect depends on beverage type. Therefore, participants were assigned to one of four groups. One group was presented with an image of a wine bottle that included an HWL (Fig. 2b), and one group was presented with an image of a vodka bottle that included an HWL (Fig. 2d). One group was presented with an image of a wine bottle without an HWL (Fig. 2a), and one group was presented with an image of a vodka bottle without an HWL (Fig. 2c). The product labels were fictitious and had been created using Adobe InDesign. The labels contained mandatory information for wine or vodka labels, such as volume or alcohol percentage, to make them look authentic. A text-only HWL was used for the two bottles with an HWL, based on previous studies’ indication that cancer-related messages were highly effective (see e.g., [13, 21]). The HWL text read, “Alcohol causes deadly liver cancer.”

Following their presentation with a bottle image, participants were asked three questions about their perceived risk of regular wine or vodka consumption, respectively. They were asked to move a slider to indicate their response. The sliders had no grid lines and no indication of values. The labels on the left and right of the sliders were adapted to each question. Participants’ ratings using the sliders were coded with values between 0 and 100. The questions were: “How do you estimate your personal risk if you consume this wine (or vodka) regularly?” (*low* [0] to *high* [100]); “*How do you estimate the impact on your health if you consume this wine (or vodka) regularly?*” (*negative* [0] to *positive* [100]); and ”How likely is it that you will suffer from negative consequences if you consume this wine (or vodka) regularly?” (*very unlikely* [0] to *very likely* [100]). The three questions’ order was randomized. Since regular consumption may differ among consumers, we did not specify how often *regularly* meant.

Therefore, participants’ risk statements referred to their respective definitions of *regular consumption*. Participants who had stated that they drank wine but not spirits were assigned to one of the two wine groups, indicated with a dotted arrow in Fig. 1. Participants who had stated that they drank spirits but not wine were assigned to one of the vodka groups, indicated with a dashed arrow in Fig. 1. All other participants (*n* = 392) were randomly assigned to one of the four groups. Principal component analysis (PCA) was conducted to determine whether the three risk items loaded on one factor. The risk scale’s explained variance of 63% and Cronbach’s α of 0.71 indicated good reliability. The mean of the three risk items was calculated and will be called *mean perceived risk* in the remainder of this paper.

#### Acceptance of health warning labels

After participants had stated their perceived risk associated with their respective products’ consumption, we assessed how acceptable they found the depiction of HWLs on wine or vodka bottles, respectively. Therefore, the two groups that had been presented with images of wine bottles (with and without an HWL) were presented with the image of a wine bottle that included an HWL at this stage of the experiment. They were asked, “Are you for or against the depiction of such labels on wine bottles?” Similar to the previous questions, this question used a slider with no gridlines or indication of value and the labels *against* (0) and *for* (100). Furthermore, we asked participants, “How acceptable do you find the HWL depicted on the bottle?” The slider for this question was labeled *unacceptable* (0) to *acceptable* (100). For the experimental groups that had been presented with images of vodka bottles, the same procedure was followed, but their questions evaluated vodka bottles with an HWL, rather than wine bottles. Since the two variables highly correlated (*r* = 0.85, *p* < 0.001) and the scale had good reliability (Cronbach’s *α* = 0.92), the mean of the two items was calculated to use in further analyses.

### Additional variables

The study assessed several constructs to further investigate consumers’ perceptions of HWLs on wine and vodka bottles, particularly the rejection of HWLs, the perceived effectiveness of such labels, social norms about drinking wine or vodka, and these beverages’ perceived positive health effects. Additionally, participants’ perceived benefits of drinking alcohol generally and their individualistic values were measured (Table 1). All constructs were measured using several items. Participants were asked to indicate their agreement using a seven-point Likert-type scale, ranging from (1) *I fully disagree* to (7) *I fully agree*. Only the extreme points of the scale were verbally anchored. Principal component analyses were conducted separately for each of the constructs to reduce the number of items. The items’ factor loadings, as well as the constructs’ explained variance and the reliability, are presented in Table 1. Four items had factor loadings below 0.6 and were excluded. For reliability, a Cronbach’s alpha and composite reliability value equal to or higher than 0.70 was considered acceptable. For the remaining items, means were calculated for each construct to support our analyses.

**Table 1:** Factor loadings of scale items reproduced by individual PCAs for each construct

Construct	Factor loading	Explained variance	Cronbach’s α	Composite reliability
**Rejection of health warning labels (HWLs)**		75.0%	0.83	0.90
“The depicted warning label is exaggerated.”	0.89			
“The depicted warning label is manipulative.”	0.84			
“The depicted warning label bothers me.”	0.87			
**HWLs’ perceived effectiveness**		51.3 %	0.89	0.93
“The depicted warning label causes people to drink less alcohol.”	0.87			
“The depicted warning label is effective.”	0.86			
“The depicted warning label leads people to rethink their alcohol consumption.”	0.86			
“The depicted warning label is helpful to reduce alcohol consumption in society.”	0.89			
**Social norms**		65.7 %	0.91	0.93
“If you have visitors, it is rude not to offer wine (or vodka).”	0.76			
“A special occasion comes with drinking a glass of wine (or vodka).”	0.87			
“It is normal to toast with wine (or vodka) in front of children.”	0.71			
“Drinking a glass of wine (or vodka) after work with colleagues is normal.”	0.79			
“A nice dinner includes a glass of wine (or vodka).”	0.90			
“Celebrating something comes with toasting with a glass of wine (or vodka).”	0.90			
“There is nothing unusual about drinking a glass of wine (or vodka) during the week.”	0.71			
**Positive health effects of wine (or vodka)**		58.7 %	0.82	0.88
“Moderate consumption of wine (or vodka) is healthy.”	0.86			
“Wine (or vodka) consumption prevents cardiovascular disease.”	0.81			
“If you only consume small amounts of wine (or vodka), you can drink every day.”	0.85			
“Wine (or vodka) consumption is only dangerous if you get drunk.”	0.68			
**Benefits of drinking alcohol**		62.6 %	0.88	0.91
“Alcohol facilitates contact with peers.”	0.83			
“When drinking alcohol, you have more fun.”	0.83			
“Alcohol helps you relax.”	0.77			
“Alcohol facilitates sexual encounters.”	0.78			
“Alcohol makes it easier to handle stress.”	0.83			
“Alcohol gives people something to do.”	0.69			
**Individualistic values**		49.5 %	0.78	0.84
“The government should do more to advance society’s goals, even if that means limiting the freedom and choices of individuals.” (recoded)	0.67			
“It’s not the government’s business to try to protect people from themselves.”	0.65			
“The government intervenes far too much in our everyday lives.”	0.76			
“Sometimes, the government needs to make laws that keep people from hurting themselves.” (recoded)	0.69			
“The government should stop telling people how to live their lives.”	0.81			

To determine the rejection and perceived effectiveness of HWLs, participants were presented with an image of a wine or vodka bottle that included an HWL, depending on their experimental group. Social norms about drinking and perceived positive health effects were measured separately for wine and vodka. Accordingly, participants in the wine groups were asked about social norms about drinking wine and about wine’s perceived positive health effects, while the vodka groups were asked the same questions but about vodka, rather than wine*.*

If a consumer perceives many benefits to drinking alcohol, such as having more fun, this perspective may affect the perceived risk of alcohol consumption. Therefore, we adapted a scale by Creyer, Kozup [14] to assess the perceived benefits of drinking alcohol without referring to any specific type of alcohol (Table 1).

The acceptance of HWLs on alcohol containers may, further, be influenced by consumers’ opinions of public authorities’ legitimacy in restricting individual rights. Therefore, we used part of a scale developed by Kahan, Jenkins-Smith [30] to assess individualistic values. The original scale comprised two subscales measuring, respectively, people’s preferences for social order in terms of social class, race, and gender (the hierarchy subscale) and the social order in terms of individual rights and restrictions for common wellbeing (the individualism subscale). Since only the individualism subscale was deemed important in the present research context, we only used the items in this subscale (Table 1). A higher mean indicates that someone has more individualistic values and opposes governments’ restrictions of individual rights. A lower mean, accordingly, means that someone supports public authorities’ interventions.

### Data analysis

2 × 2 analyses of variance (ANOVAs) with the independent variables *HWL group* (two levels) and *beverage type* (two levels) were used to estimate the main effects and interaction effects on perceived risk, acceptance, and rejection. Furthermore, separate linear regressions for wine and vodka were used to estimate the predictors of perceived risk and the rejection of HWLs on wine and vodka bottles, respectively. The explanatory variables of perceived effectiveness of HWLs, social norms, positive health effects, perceived benefits of drinking alcohol, and individualistic values were analyzed for the four experimental groups using 2 × 2 ANOVAs.

## Results

The four experimental groups (for wine and vodka bottles with and without HWLs) did not differ in terms of gender, *χ*^2^(3) = 0.78, *p* = 0.855, *φ* = 0.039*,* education, *F*(3, 502) = 0.95, *p* = 0.418, partial *η*^*2*^ = 0.006, or alcohol consumption, *F*(3, 502) = 0.35, *p* = 0.790, partial *η*^*2*^ = 0.002, suggesting that our experiment’s randomization succeeded. The four groups did differ, however, in age, *F*(3, 502) = 6.59, *p* < 0.001, partial *η*^*2*^ = 0.038. The vodka groups with an HWL (*M* = 43, *SD* =15) and without an HWL (*M* = 44, *SD* =15) had a lower average age than the wine groups with an HWL (*M* = 50, *SD* =17) and without an HWL (*M* = 49, *SD* =16). This difference may have occurred because participants were asked how often they consumed spirits such as gin, whiskey, or vodka. These spirits are often used as ingredients in cocktails, which are mainly consumed by younger people [33]. The difference in age between the wine and vodka groups was considered in the following analyses.

In investigating HWLs’ influence on risk perceptions for wine and vodka, this study found that HWL did not significantly affect mean risk perceptions.^2^ The inclusion of an HWL on an alcohol container did not increase perceived personal risk, *F*(1, 502) = 0.01, *p* = 0.920, partial *η*^*2*^ < 0.001. However, beverage types significantly affected perceived personal risk, *F*(1, 502) = 127.66, *p* < 0.001, partial *η*^*2*^ = 0.203. No significant interaction term was observed, *F*(1, 502) = 0.00, *p* = 0.979, partial *η*^*2*^ < 0.001. Participants in the wine groups perceived, on average, significantly less risk (*M* = 47, *SD* = 21) than participants in the vodka groups (*M* = 66, *SD* = 19). Therefore, participants who had been presented with an image of a wine bottle perceived a lower personal consumption risk, a less negative health impact for regular consumption, and a lower likelihood of suffering negative consequences due to regular consumption than participants who had been presented with an image of a vodka bottle.

We, therefore, rejected our hypothesis that HWLs increase consumers’ perceived alcohol consumption risk. However, we accepted our hypothesis that consumers’ perceived vodka consumption risk exceeds their perceived wine consumption risk.

Since wine and vodka seem to elicit different levels of perceived risk, two linear regressions were conducted to assess how different factors influenced risk perceptions about regular consumption (Table 2). *Mean perceived risk* was the dependent variable. The predictors were *HWL group*, *social norms*, *perceived positive health effects*, *perceived benefits of drinking alcohol*, *wine or vodka consumption*, respectively, *gender*, *age*, and *education*. Both the model for wine consumption, *F*(8, 247) = 8.94, *p* < 0.001, adjusted *R*^*2*^ = 0.20, and the model for vodka consumption were significant *F*(8, 241) = 7.40, *p* < 0.001, adjusted *R*^*2*^ = 0.17.

**Table 2:** Linear regression of the perceived risk of wine and vodka consumption

	Wine (*n* = 255)				Vodka (*n* = 250)			
	Unstandardized B [95% CI]	SE (B)	Beta	*t*	Unstandardized B [95% CI]	SE (B)	Beta	*t*
Constant	56.28 [28.04, 84.52]	14.34		3.92**	75.00 [46.94, 103.06]	14.25		5.26**
Health warning label (HWL) group ^b^	0.48 [-4.06, 5.03]	2.31	0.01	0.21	0.44 [-3.88, 4.75]	2.19	0.01	0.20
Social norms ^a^	-1.29 [-3.31, 0.74]	1.03	-0.09	-1.25	-2.81 [-5.68, 0.06]	1.46	-0.16	-1.93
Positive health effects ^a^	-6.04 [-8.11, -3.96]	1.05	-0.39	-5.72**	-4.97 [-7.52, -2.42]	1.29	-0.30	-3.84**
Benefits of drinking alcohol	1.67 [-0.03, 3.36]	0.86	0.12	1.94	1.27 [-0.62, 3.15]	0.96	0.09	1.32
Alcohol consumption ^a^	-0.08 [-0.25, 0.10]	0.09	-0.05	-0.88	-0.11 [-0.49, 0.27]	0.19	-0.04	-0.57
Gender ^c^	3.58 [-1.06, 8.23]	2.36	0.09	1.52	3.74 [-0.77, 8.24]	2.29	0.10	1.63
Age	-0.04 [-0.19, 0.12]	0.08	-0.03	-0.47	-0.12 [-0.27, 0.03]	0.08	-0.10	-1.62
Education	0.71 [-1.72, 3.14]	1.23	0.03	0.58	-1.00 [-3.36, 1.36]	1.20	-0.05	-0.83

* *p* < 0.05, ** *p* < 0.01

^a^ These variables were beverage-specific. For example, *social norms* referred to social norms about drinking wine for the wine groups and social norms about drinking vodka in the vodka groups

^b^ Dummy-coded HWL group: 0 = no HWL, 1 = with an HWL

^c^ Dummy-coded gender: 0 = male, 1 = female

For both wine and vodka, the only significant predictor of perceived risk was the perceived positive health effects of wine or, in the vodka groups’ case, vodka. This finding shows that the more someone perceives health benefits from the consumption of wine or vodka, the lower their perceived consumption risk.

We further examined the acceptance of HWLs using a 2 × 2 ANOVA with *acceptance* as the dependent variable and *HWL group* and *beverage type* as independent variables. A significant effect was found for *beverage type*, *F*(1, 502) = 76.02, *p* < 0.001, partial *η*^*2*^ = 0.132. HWLs were perceived as more acceptable on vodka bottles (*M* = 61, *SD* = 31) than on wine bottles (*M* = 36, *SD* = 33). Similar to our earlier finding that exposure to an alcohol container with an HWL in assessing perceived consumption risk did not alter the acceptance of HWLs, *F*(1, 502) = 0.29, *p* = 0.588, partial *η*^*2*^ = 0.001, the interaction between *HWL group* and *beverage type* had no effect. Therefore, we accepted our hypothesis that HWLs are perceived as more acceptable on vodka bottles than on wine bottles.

The rejection of HWLs was found to be an important indicator of consumers’ perception of HWLs. Our results show that the rejection of HWLs was significantly higher for wine compared to vodka, *F*(1, 501) = 58.70, *p* < 0.001, partial *η*^*2*^ = 0.105. Exposure to an HWL during the experiment did not affect participants’ rejection of HWLs, *F*(1, 501) = 1.44, *p* = 0.230, partial *η*^*2*^ = 0.003, for either wine or vodka. To investigate the influences on participants’ rejection of HWLs, we again conducted two separate linear regressions for wine and vodka. The predictors were *perceived effectiveness*, *social norms*, *perceived positive health effects of wine and vodka*, *perceived benefits of drinking alcohol*, *individualistic values*, *alcohol consumption*, *gender*, *age*, and *education* (Table 3).

**Table 3:** Linear regression for the rejection of health warning labels (HWLs) on wine and vodka bottles

	Wine (*n* = 255)				Vodka (*n* = 250)			
	Unstandardized B [95% CI]	SE (B)	Beta	*t*	Unstandardized B [95% CI]	SE (B)	Beta	*t*
Constant	2.39 [0.07, 4.70]	1.18		2.03	1.42 [-0.88, 3.73]	1.17		1.22
HWLs’ effectiveness^a^	-0.38 [-0.52, -0.24]	0.07	-0.32	-5.41**	-0.41 [-0.53, -0.28]	0.06	-0.36	-6.31**
Social norms^a^	0.17 [0.01, 0.33]	0.08	0.15	2.15*	0.20 [-0.03, 0.43]	0.12	0.13	1.73
Positive health effects^a^	0.22 [0.05, 0.39]	0.09	0.17	2.60*	0.19 [-0.01, 0.40]	0.10	0.13	1.87
Benefits of drinking alcohol	0.13 [0.00, 0.27]	0.07	0.12	1.94	0.11 [-0.04, 0.26]	0.08	0.09	1.43
Individualistic values	0.20 [0.04, 0.36]	0.08	0.15	2.52*	0.31 [0.16, 0.45]	0.07	0.24	4.13**
Alcohol consumption^a^	0.00 [-0.01, 0.01]	0.01	-0.01	-0.14	0.00 [-0.03, 0.03]	0.02	-0.01	-0.15
Gender^b^	0.01 [-0.35, 0.38]	0.18	0.00	0.07	0.08 [-0.28, 0.43]	0.18	0.02	0.43
Age	0.00 [-0.01, 0.01]	0.01	0.02	0.41	0.01 [0.00, 0.02]	0.01	0.08	1.42
Education	0.07 [-0.12, 0.26]	0.10	0.04	0.71	0.03 [-0.16, 0.21]	0.10	0.01	0.27

* *p* < 0.05, ** *p* < 0.01

^a^ These variables referred to beverage types. For example, participants in the wine groups were asked about HWLs’ effectiveness on wine bottles, whereas participants in the vodka groups were asked about HWLs’ effectiveness on vodka bottles

^b^ Dummy-coded gender: 0 = male, 1 = female

The model was significant for both wine, *F*(9, 246) = 11.75, *p* < 0.001, adjusted *R*^*2*^ = 0.28, and vodka, *F*(9, 240) = 12.76, *p* < 0.001, adjusted *R*^*2*^ = 0.30. The strongest predictor in both models was *perceived effectiveness*, followed by *individualistic values*. In the wine case, *perceived health benefits* and *social norms of drinking* were additional significant predictors. The models explained 28% of participants’ rejection of HWLs in the wine groups and 30% of the corresponding rejection in the vodka groups.

In other words, the more a participant perceived HWLs as ineffective and opposed government restrictions of individual rights, the higher their rejection of HWLs. For participants in the wine groups, beliefs that wine consumption offers positive health effects and that drinking wine is part of social norms further increases the rejection of HWLs.

These results show that consumers seem to have different associations with wine compared to vodka, which influences how they react to HWLs on these beverages’ containers. HWLs on wine bottles (*M* = 2.9, *SD* = 1.4) were perceived to be significantly less effective, *F*(1, 503) = 4.69, *p* = 0.031, partial *η*^*2*^ = 0.009, than HWLs on vodka bottles (*M* = 3.3, *SD* = 1.4). Furthermore, the social norms of drinking, as well as its perceived positive health effects, were found to differ between wine and vodka. On average, the wine groups (*M* = 4.4, *SD* = 1.5) expressed higher scores for social norms, *F*(1, 503) = 337.06, *p* < 0.001, partial *η*^*2*^ = 0.402, than the vodka groups (*M* = 2.3, *SD* = 1.1), indicating that drinking wine aligns more with social norms than vodka does—for example, when entertaining guests or on special occasions. Moreover, wine (*M* = 4.4, *SD* = 1.3) was perceived to offer more beneficial health effects, *F*(1, 503) = 174.95, *p* < 0.001, partial *η*^*2*^ = 0.259, than vodka (*M* = 2.9, *SD* = 1.1). The experimental groups with and without HWLs did not differ in terms of perceived effectiveness, social norms, or perceived positive health effects, and the interaction between HWL groups and beverage types was also insignificant.

## Discussion

Our results suggest that the perceived risk of regular alcohol consumption is determined by the type of alcoholic beverage but not altered by bottles’ inclusion of an HWL. Increasing risk perceptions has been suggested to effectively influence drinking behavior [34]. Our study indicates that HWLs may not be the right approach for such interventions. We could not replicate the findings of an earlier study in which the same kind of cancer HWLs on wine bottles increased perceived consumption risks compared to HWL-free bottles [22]. The reason for this difference may be that the previous study assessed the perceived risk of developing cancer, while the present study measured both personal risk and the likelihood of suffering negative consequences due to regular consumption. Therefore, HWLs may be able to raise risk perceptions of a specific threat but not perceived consumption risks in general.

Earlier studies about HWLs found that HWLs may provoke negative emotional arousal, stronger intentions to reduce drinking, slower consumption, or avoidance [18, 21, 25, 27]. While previous research has found that HWLs affect consumers in several different ways, the current study yielded no such findings, perhaps because the previous studies used different HWLs (e.g., image-and-text labels) [21, 25, 27] or young-adult or adolescent participants, rather than adult participants [12, 16, 18, 23, 35–38]. Wine is mainly consumed by older consumers, and wine HWLs’ effect on younger consumers may not be comparable to their effect on older consumers. Furthermore, some studies have collected data in countries such as the United States or Australia with different alcohol cultures [14, 17, 28]. Many European countries have particularly alcohol-friendly cultures, which influence how consumers react to interventions seeking to change their drinking behavior [16].

Much research has evaluated HWLs’ design and framing to determine the greatest effect on consumers (see, e.g., [13, 38]) but neglected beverage types’ influence. The present study successfully showed that a deeply culturally rooted beverage (such as wine in Switzerland) does not elicit the same level of risk perception as a beverage without such a cultural significance (such as vodka in Switzerland), so consumption of the former is, therefore, unaffected by HWLs. Some readers might argue that such differences in perceived risk are due to the beverages’ different alcoholic strengths. A standard unit of an alcoholic drink refers to a specific amount of pure alcohol [2], so regardless of whether a person drinks a glass of wine or a small shot of vodka, the effect of the servings’ ethanol is comparable [4]. However, consumers seem to perceive different levels of consumption risk for different types of alcoholic beverages. The reason for this difference in the current study may be Swiss consumers’ associating wine with traditional drinking norms, whereas they associate spirits consumption more with adverse health effects [29, 39]. Although beverage-specific variations in risk perception have been reported [40], the probability of experiencing negative outcomes of alcohol consumption was also found to be better predicted by alcohol consumption level than preferred beverage types [39].

The notion revealed in the current study that wine is a “healthy” alcoholic beverage confirms earlier findings that people believe other beverages have more detrimental effects on health than wine [15, 41]. Consumers’ convictions about wine’s positive health effects were found to be unaffected by HWLs, but these convictions may differ between countries, depending on wine’s role in their cultures [14]. Our results suggest that health beliefs are an important driver of perceived consumption risks and how consumers react to HWLs. Policy-makers must account for consumers’ associations with different alcoholic beverages’ harmfulness or perceived positive health effects, which determine their perceived risk and—eventually—their drinking behavior [40].

The awareness of the link between alcohol consumption and disease was found to be associated with increased support for alcohol policies [6]. Consumers who are aware of the negative consequences of drinking alcohol show a lower rejection of measures such as HWLs on alcohol containers. However, Peadon et al. [34] argued that increasing consumers’ risk perceptions of drinking alcohol may be more effective than traditional awareness campaigns. That is, an alcohol policy such as including HWLs on alcohol containers may be more effective in reducing consumers’ alcohol intake if it increases the perceived risk of consumption as opposed to being used as a mean to increase their awareness that drinking alcohol can cause cancer. Our findings indicate that consumers may not be aware of the link between alcohol and health risks and that HWLs may not prove effective in enhancing the perceived risk of consumption. Therefore, campaigns that correctly inform people about alcohol’s influence on their health and address the “wine is healthy” attitude may be more accepted and successfully increase consumers’ perceived risk compared to interventions such as HWLs [42]. Addressing such health beliefs may be an effective step in raising awareness of the risks associated with drinking alcohol.

The current study’s findings indicate that a lack of (perceived) effectiveness is a major driver of people’s HWLs rejections. Previous research found that consumers perceive warning labels as ineffective and do not believe their drinking behavior would change after HWLs were implemented [16, 28]. Reynolds et al. [26] suggest that emphasizing the effectiveness of an intervention (such as a warning label) could increase its acceptability. Although participants in the current study perceived HWLs as more effective on vodka bottles than on wine bottles, our study does not provide evidence suggesting that HWLs raise risk perceptions and may, therefore, effectively alter drinking behavior.

Earlier work found that the acceptance of an intervention may not depend on intrusiveness [43]. Furthermore, food labels have been found to be a well-accepted policy intervention [44]. The present study found that, when consumers are confronted with an intervention in a realistic scenario—such as confronting HWLs on alcohol containers—such interventions may be perceived as too intrusive. This perception is especially likely in the case of culturally important products, such as wine in Switzerland, and acceptance of such interventions may be low. Individualistic values were found to be an important driver of consumers’ HWL rejection, in line with an earlier study that found the acceptance of wine HWLs to be lower among consumers with individualistic values [22]. These cultural values have been reported to affect support for policies in other contexts, such as climate change [45].

Drinking alcohol is customary in many European countries. The social norms of drinking and its high acceptance in western societies have grown historically, supported by the alcohol industry’s strong political influence [10]. The perceived pleasure and benefits that people associate with wine drinking may be so high that wine’s risks are considered “reasonable,” resulting in consumers’ rejection of approaches such as HWLs to warn them about alcohol consumption’s potential negative effects. Consequently, governments face little pressure to change their alcohol policies.

### Limitations and implications for future research

This study faced several limitations. Participants were assigned to either vodka or wine groups. Therefore, we did not determine whether a low acceptance and strong rejection of wine HWLs could also be found if the wine-group participants had also evaluated vodka HWLs. We did not include beer, a popular alcoholic beverage in Switzerland that accounts for a large part of the consumed alcohol, due to the absence of health benefits associated with beer consumption. We do not know whether the perceived risk of beer would be more similar to the perceived risk of wine or of vodka. Moreover, we used a single, text-only HWL in this study and risk perception was assessed after short exposure to the HWL. We did not assess what *regular consumption* meant to participants, so their risk perceptions may have referred to different consumption levels. Additionally, this study was conducted in Switzerland, limiting our findings’ generalizability. Consumers in other countries with different alcohol cultures and preferred beverages may not react the same way that this study’s Swiss participants reacted. Finally, this study used an online format to assess respondents’ perceptions of HWLs. However, we do not know how such labels would be perceived and reacted to in real-life situations, such as restaurants or grocery stores. Repeated and widespread exposure to HWLs may result in different reactions and behavior of consumers.

Despite these limitations, this study’s findings offer several important implications. The study’s HWLs, with a text-only cancer warning statement, did not increase respondents’ risk perceptions, and health beliefs were found to be major predictors of risk perceptions. Therefore, future studies should investigate whether HWLs may effectively raise risk perceptions of drinking alcohol in societies with different alcohol cultures, where beliefs about wine consumption’s positive health effects are less abundant. This study found that risk perceptions for alcohol consumption were higher for vodka than wine. Researchers should, accordingly, examine how different associations for various beverages influence behavior and how our findings could be used to reduce the negative outcomes of alcohol consumption through communication. For example, researchers could use a specific alcoholic beverage, such as vodka, to inform about the risk of consuming one standard unit of a drink. Policy-makers should address the widespread belief that wine consumption can offer positive health effects so that consumers can correctly assess their consumption risk and understand that alcohol’s damage primarily stems from consumption patterns, not beverage types.

## Conclusion

The present study investigated the acceptance of HWLs and their effectiveness on wine and vodka bottles as a potential policy intervention to increase consumers’ perceptions of alcohol consumption risks. We found that risk perceptions did not increase when alcohol containers included an HWL but, rather, were determined by the beverage types that consumers considered drinking. The risk of drinking wine was perceived to be lower than the risk of drinking vodka. Consumers who believe in positive health effects from drinking wine or vodka had lower risk perceptions and rejected HWLs. The acceptance of HWLs was higher among participants who thought HWLs were effective but lower among participants with individualistic values who refused governmental restrictions to individual rights. Drinking wine is part of social norms in Switzerland, but drinking vodka is less socially normative in this country. This difference is reflected in participants’ negative reaction toward and low acceptance of HWLs on wine bottles. Therefore, policy-makers must account for such interventions’ effectiveness and—more importantly—acceptance possibly varying, depending on beverage types. Europe’s drinking culture is linked to specific beverages, and this study’s findings may not be reproduced through similar assessments in other regions where wine is less abundant. Risk perception is important in determining the degree to which someone engages in a risky behavior, such as alcohol consumption, but it may not be affected by interventions such as HWLs on alcohol containers. This study’s findings add to the knowledge about HWLs and risk perception related to alcohol consumption. Furthermore, this study has highlighted the importance of beliefs about alcohol consumption’s positive health effects and their influence on perceptions of risky drinking behaviors.

## Data Availability

Anonymized data have been deposited in OSF (Open Science Framework), https://osf.io/d5fbc/ [46].

## Footnotes

1 A standard drink or unit of alcohol (100–120 ml red wine or 30–40 ml of high strength spirit like vodka) contains roughly 10–12 grams of pure alcohol [32].

2 Since the experimental groups differed by average age, a univariate analysis of covariance (ANCOVA) was conducted using *age* as a covariate. The variable *age* was significant, *F*(1, 501) = 6.25, *p* = 0.013, partial *η*^*2*^ = 0.012, but the results for the main and interaction effects were the same as without the covariate. Therefore, we reported our ANOVA results. Additionally, univariate ANOVAs were conducted separately for each of the three risk items. The main and interaction effects, or the lack thereof, were the same as for the *mean risk perception* variable.
